# Complete Genome Sequences of Two T4-Like Escherichia coli Bacteriophages

**DOI:** 10.1128/genomeA.00586-18

**Published:** 2018-06-28

**Authors:** Ana Rita Costa, Stan J. J. Brouns, Franklin L. Nobrega

**Affiliations:** a Department of Bionanoscience, Kavli Institute of Nanoscience, Delft University of Technology, Delft, The Netherlands; b Department of Microbiology, Wageningen University and Research Centre, Wageningen, The Netherlands

## Abstract

Bacteriophages and their proteins have potential applications in biotechnology for the detection and control of bacterial diseases. Here, we describe the sequencing and genome annotations of two strictly virulent Escherichia coli bacteriophages that may be explored for biocontrol strategies and to expand the understanding of phage-host interactions.

## GENOME ANNOUNCEMENT

Escherichia coli is a commensal Gram-negative microorganism inhabiting the gastrointestinal tract of humans and animals, but it is also known for causing fatal infections (1–3). With the emergence of antibiotic-resistant bacteria, alternatives to antibiotics are lacking; bacteriophages and their derived proteins are a promising solution (4, 5).

Here, we isolated phages from ditch samples (Wageningen, The Netherlands) using a multihost approach of 32 E. coli strains (including O157:H7) and propagation in E. coli BL21. Both phages belong to the Myoviridae family and have broad lytic activity (in 15/32 strains for vB_CEB_NBG1 and 12/32 strains for vB_CEB_NBG2). Phage DNA was extracted as previously described (6). Sequencing was performed by Nucleomics Core using the next-generation sequencing (NGS) Illumina MiSeq platform and NEBNext Ultra DNA library prep kit. Sequencing reads (≈300 bp) with more than 100-fold coverage were *de novo* assembled using CLC Genomics Workbench version 7.0 (Aarhus, Denmark) and manually inspected. Annotation was performed using the RAST server (7), followed by manual screening of all predicted proteins against the NCBI protein database using BLASTp (8) and a Pfam domain search (9). tRNAs, promoters, and terminators were predicted with tRNAscan-SE version 2.0 (10), Geneious version 9.1.3 using motif TTGACAN(15,18)TATAAT with a maximum of one mismatch, and ARNold (11), respectively. The genome packaging strategy was predicted by phylogenetic analysis of the large terminase subunit (12).

Phages vB_EcoM_NBG1 and vB_EcoM_NBG2 have linear double-stranded DNA, with genome sizes of 168,869 bp and 166,083 bp (33.8% homologous), 2 (Arg and Met) and 10 (Gln, Leu, Gly, Pro, Ser, Thr, Met, Tyr, Asn, and Arg) tRNAs, 8 promoters each, and 27 and 20 Rho-independent terminators, respectively. Their GC contents of 37.7% and 35.4% are lower than that of E. coli (≈50%) (12).

Phages vB_EcoM_NBG1 and vB_EcoM_NBG2 have 269 and 261 predicted open reading frames (ORFs), of which 125 (46.5%) and 134 (51.3%) could be assigned a function, respectively. None of the predicted proteins exhibit homology toward virulence factors, integration-related proteins, or antibiotic resistance determinants; no genomic markers were found indicating a temperate lifestyle. These genetic features make both phages suitable candidates for phage therapy. Also, proteins were identified with exploratory interest, such as tail fibers (genes 165, 173, 216, 246, and 248 to 250 of vB_EcoM_NBG1 and genes 148, 200, 201, and 234 to 238 of vB_EcoM_NBG2), endolysins (gene 124 of vB_EcoM_NBG1), holins (genes 251 of vB_EcoM_NBG1 and 239 of vB_EcoM_NBG2), spanin complex (genes 228 and 229 of vB_EcoM_NBG1 and genes 212 and 213 of vB_EcoM_NBG2), and tail-associated lysozymes (gene 156 of vB_EcoM_NBG1 and genes 118 and 139 of vB_EcoM_NBG2).

Phylogenetic analysis of the large terminase subunit suggests both phages use a T4-like mechanism of headful packaging, with no preferred packaging signal (12). As this implies random phage termini, phages were zeroed using phage T4 as a reference.

Comparative genomics revealed that phage vB_EcoM_NBG1 is closely related to Escherichia phage APCEc01 (accession number NC_029091), sharing 98% identity over 98% of its sequence, whereas phage vB_CEB_NBG2 is closely related to Escherichia phage PEC04 (accession number KR233165), with 97% identity over 95% of its sequence. The genome comparisons revealed regions with pronounced differences located mainly in the tail fiber proteins, which likely confer the phages a distinct host lytic spectrum.

### Accession number(s).

The genome sequences have been deposited in GenBank under the accession numbers MH243438 (vB_EcoM_NBG1) and MH243439 (vB_EcoM_NBG2).
